# Hormonal treatment for men with Non-obstructive Azoospermia: too many rationales, too little data

**DOI:** 10.1590/S1677-5538.IBJU.2022.99.13.1

**Published:** 2022-04-14

**Authors:** Filipe Tenorio Lira

**Affiliations:** 1 Andros Recife Recife PE Brasil Andros Recife, Recife, PE, Brasil

## COMMENT

Non-obstructive azoospermia (NOA) is the most severe form of male infertility, and despite all the research efforts, the last (and only) breakthrough in the management of NOA, the development of testicular sperm extraction techniques and intracytoplasmic sperm injection (ICSI), happened three decades ago and only benefit approximately half of the affected men (1). Because an etiological diagnosis is not possible in most cases, more often than not, men with NOA are managed using a “syndromic” approach that usually ends in a testicular sperm extraction procedure. The most challenging cases are those men that failed a testicular sperm extraction procedure, which are often offered sperm donation or adoption

In the current paper, Laursen et al. (2) described the first case series of men with NOA that failed a diagnostic testicular sperm extraction and underwent hormonal stimulation with recombinant gonadotropins. The participants were treated with recombinant human chorionic gonadotropin (hCG) and recombinant follicle-stimulating gonadotropin (FSH). The goal was to increase testosterone to the upper limit levels while keeping FSH in the normal range. After treatment for a mean duration of 10 months, the authors reported that 25% of the participants had viable sperm in the ejaculate and another 25% had viable sperm in a second testicular sperm retrieval. Despite the limitations of such small case series, the authors should be commended to have published their results, since this topic is frequently discussed among male infertility specialists, but rarely addressed via scientific publications.

Recombinant gonadotropins have been shown to have less batch-to-batch variation and contaminants when compared to preparations from purified urine (3). In addition, these medications are marketed in prefilled pens that allow dose adjustment and are easier to use. However, from female studies, it still unclear if these advantages translate into better reproductive outcomes (4). Thus, studies assessing the efficacy and safety of these preparations in the management of male infertility are welcomed. Conversely, the exciting results reported by the authors may be derived from the high proportion of men with hypospermatogenesis and late maturation arrest included in the study. It is known that these two histological patterns cofer good prognosis for both hormonal stimulation and salvage sperm retrieval (5, 6). Not surprisingly, only men with these histological patterns benefited from the treatment in the current study. Moreover, the long duration of the treatment may also explain the good results. However, the duration and the costs of the recombinant medications may restrict the applicability this hormonal stimulation protocol.

Due to the still suboptimal results of testicular sperm extraction procedures, male infertility specialists often try to improve any residual spermatogenesis using several medical treatments such as antioxidants, vitamins, and “optimizing” the sexual hormones levels. Nevertheless, the evidence level for most of these treatments are low (7). Besides, it is still unclear what are the optimal levels of such hormones for sperm production, and most cut-offs found in the literature are arbitrary (8). More confusion is added by the large heterogeneity of phenotypes displayed by men with NOA, thus, it is obvious that different patients will require different levels of sexual hormones, a classic example of “one size does not fit all”

Several protocols of “hormonal stimulation” have been proposed for use in this scenario. Some of these protocols include gonadotropins to increase testosterone above a certain cut-off levels, others consist in increasing FSH to supraphysiologic levels, whereas some even advocate the combined use of GnRH antagonists or testosterone with gonadotropins to bring FSH to “normal” levels in those men with high baseline FSH (6, 9, 10). However, most of the studies included a small number of participants and the results had disputable clinical significance. Furthermore, if these protocols are used in men with NOA before their first attempt of sperm retrieval, without knowing their predominant testicular histological pattern, we risk disturbing their endogenous hormonal milieu that might be responsible to sustain small foci of active spermatogenesis or prescribing a futile treatment.

Therefore, before the widespread use of “hormonal stimulation” in NOA cases, there are several questions yet to be answered: What are the optimal sexual hormone levels for spermatogenesis in a specific individual? Is it necessary to achieve these levels before the first attempt of sperm retrieval? What medications and how long should them be used? To clarify this topic, well designed randomized controlled trials are urgently needed as well as large observational real-life studies.
